# Supplementary material for: Unveiling connectivity patterns of railway timetables through complex network theory and Infomap clustering
Source: PLoS One. 2025 Jul 18;20(7):e0328681. doi: 10.1371/journal.pone.0328681 (PMC12273951; doi:10.1371/journal.pone.0328681)
Supplement: S1 File — This file includes 3 figures (S1 Fig: Distribution of Interquantile Range within Modules; S2 Fig: Scatterplots showing the relationship between module size (Nm) and module flow for different modular structures of the Norwegian railway timetable network; S3 Fig: Sensitivity Analysis of Tc across full day, peak and off-peak time windows) and 2 tables (S1 Table: Timetable data: general input schema; S2 Table: Norwegian Timetable; first Level of clustering results). (PDF) [file pone.0328681.s001.pdf]

Supporting Information for *Unveiling connectivity patterns of railway timetables through complex network theory and Infomap clustering*

Fabio Lamanna,[1,*] Michele Prisma,[2] and Giorgio Medeossi[2]

[1] *Freelance Civil Engineer, Treviso, Italy*
[2] *Trenolab, Gorizia, Italy*

Supplementary Figures

Timetable Connectivity Index

The Timetable Connectivity Index (T_c) defined in this paper arises from further analysis of the flow distribution within modules. Our initial approach incorporated a "distribution" factor into the definition, as follows:

$$T_{c,IQR} = \frac{1}{N} \sum_{m=1}^{M} N_m F_m (1 - IQR), \tag{1}$$

where we augment the original formula with the interquartile range (IQR) of total nodes' flow (within each Module) distribution. The interquartile range is defined as the difference between the 75th and 25th percentiles of the data. By including the factor $(1 - IQR)$, we ensure that when flow variability is minimal (i.e., IQR is close to zero), its contribution to the overall index becomes negligible. Figure S1 shows the distribution of IQR values of nodes' flow within Modules for both Scenarios R24 and R33, revealing their negligible contribution. In general, it remains important to examine the flow distribution in each module when evaluating its contribution to T_c.

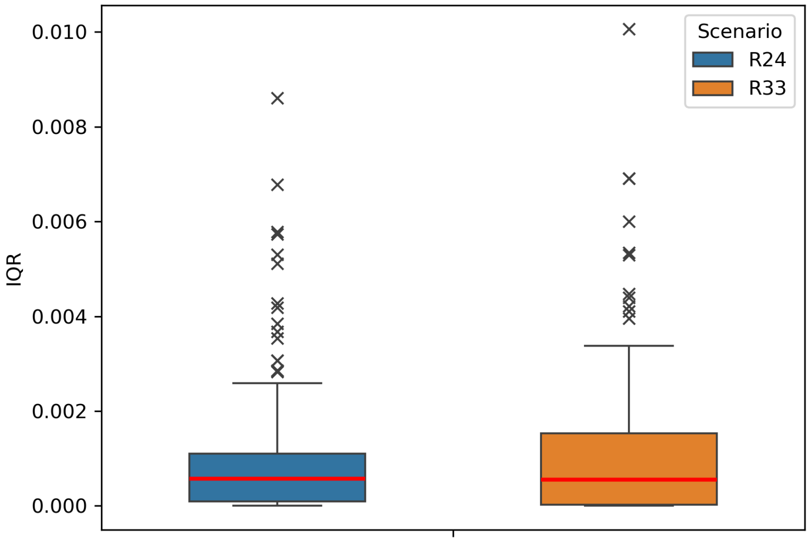

Fig S1: **Distribution of Interquantile Range within Modules.** Considering the distribution of Infomap flow within nodes in each Module (and in both Scenarios), we plot the distribution of the Interquantile Range of the values to get information about the contribution of the variability of flows to the Timetable Connectivity Index.

*Corresponding authors: fabio@fabiolamanna.it

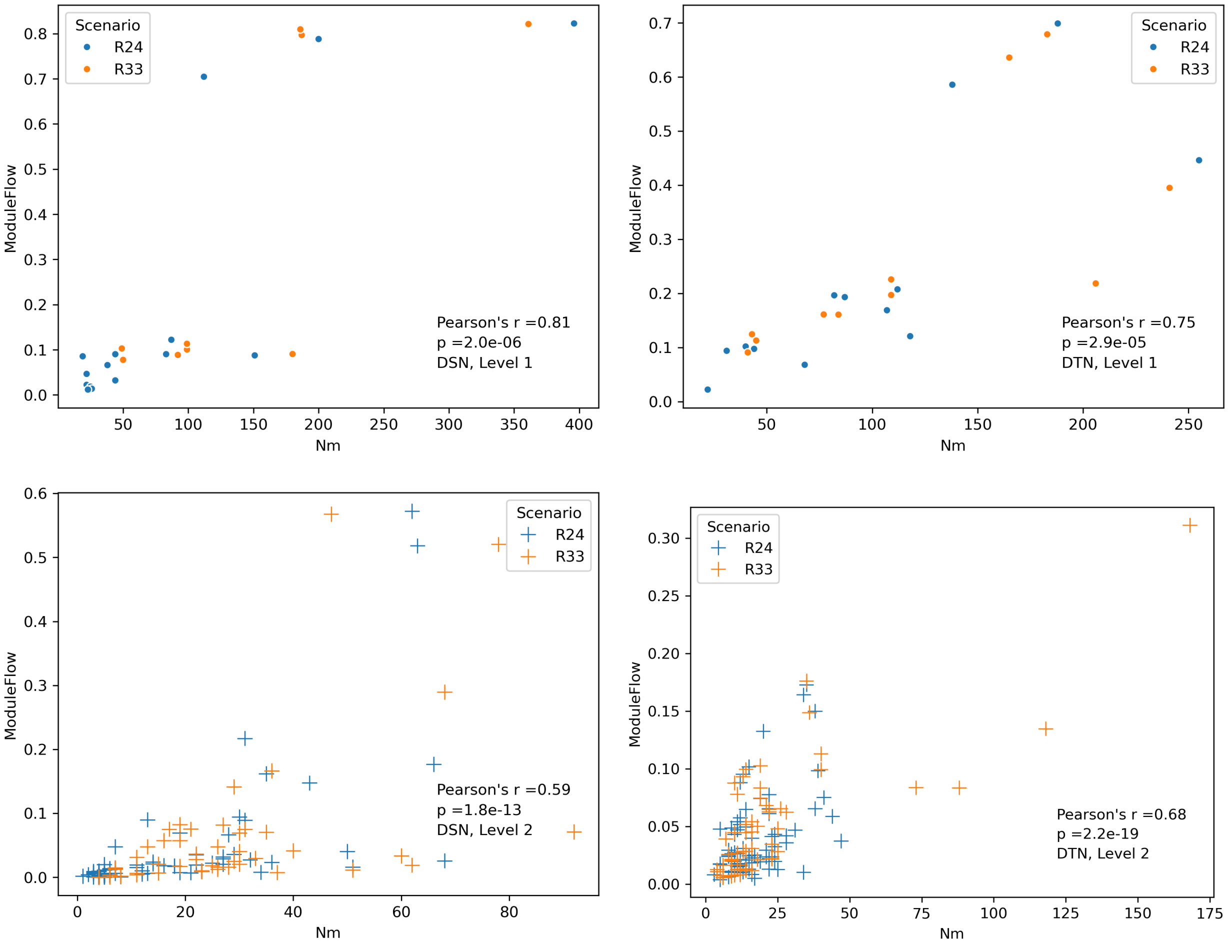

Fig S2: **Scatterplots showing the relationship between module size (N_m) and module flow for different modular structures of the Norwegian railway timetable network.** Top row: Directed Service Network (DSN) at first-level (left) and second-level (right) of clustering; bottom row: Directed Travel Time Network (DTN) at first-level (left) and second-level (right) of clustering. The Pearson correlation coefficients between module size and flow are 0.81 for DSN first-level, 0.59 for DSN second-level, 0.75 for DTN first-level, and 0.68 for DTN second-level, all with p-values $< 10^{-5}$, indicating statistically significant relationships. The first-level clustering reveals a few large modules concentrating most of the network flow, whereas the second-level clustering uncovers a finer-grained modular organization with many smaller modules carrying lower flow values. Differences between DSN and DTN highlight the contrasting structural tendencies of service-based and travel time-based connectivity, with DSN exhibiting tighter service-based aggregation and DTN reflecting more dispersed accessibility patterns.

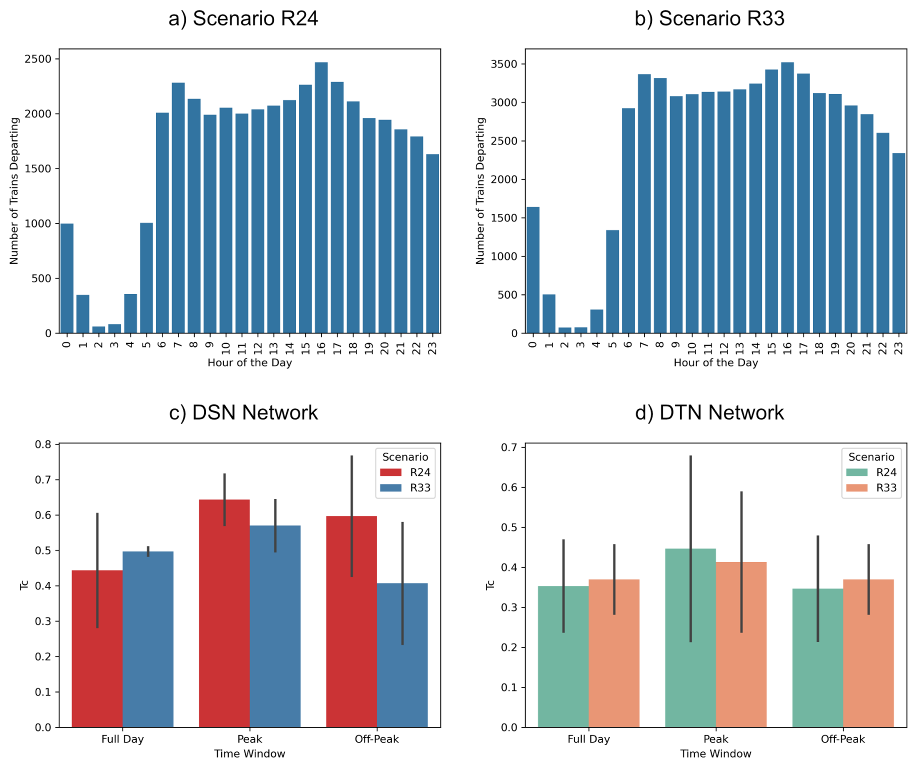

Fig S3: **Sensitivity analysis of T_c across full day, peak and off-peak time windows.** The first thing to do is to define peak and off-peak periods in our Norwegian timetable. Since we are dealing with services at a national scale, we didn't expect particular differences between peak and off-peak services in terms of number of trains running (with the exception of nighttime services). We were able to define two peaks (one in the morning between 7 and 8, one in the afternoon between 16 and 17). Those peaks are consistent in both scenarios a) R24 and b) R33 despite the higher number of trains running in the forecasted timetable. We verified also the distribution among arrival times, and they are consistent with the previously defined peak time frames. We then filter out our initial dataset getting information related to train departing in peak only hours and off-peak only hours, applying our general framework on the filtered datasets. Subfigure c) and d) show the average values of T_c in both network of analyses, for all spaces with the indication of the standard variation for each time windows and scenario. For both networks, T_c during peak periods is generally higher than in the full-day and off-peak time frames. This is due to the more concentrated services during peak hours, which typically form well-defined clusters—both in terms of service frequency and travel time. In the DSN network c), T_c is higher in the forecasted scenario (R33) when considering the full-day period. However, peak and off-peak values are lower compared to the current R24 timetable. This suggests a redistribution of services in R33 that favors peak operations and potentially prioritizes express routes, leading to reduced modular cohesion during off-peak periods. The decline in off-peak T_c may reflect a fragmentation of stop-to-stop connectivity for local services in the future timetable. Less variability is observed between the two scenarios in the DTN network d), where—despite the inclusion of faster and more frequent long-distance services in R33—the average travel time-based accessibility at the stop level declines, likely due to express services bypassing intermediate stops.

Supplementary Tables

Train number	Station	Arrival time	Departure time	Stop type
E35	KWX		08:05:00	begin
E35	VMF	08:25:54	08:25:54	pass
E35	RFK	08:44:08	08:46:08	stop
E35	KAV	09:13:31	09:15:31	stop
E35	ZJI	09:16:00		end
R53	ZJI		07:04:00	begin
R53	KWX	07:33:07	07:35:07	stop
R53	RFK	08:14:53	08:15:53	stop
R53	KAV	08:21:00		end
E42	ZJI		09:58:00	begin
E42	KAV	10:09:45	10:09:45	pass
E42	RFK	10:50:42	10:50:42	pass
E42	VMF	11:11:00		end

Table S1: **Timetable data: general input schema.** The table shows a sample timetable generated by the code available in the GitHub repository. It includes three trains operating across five stations, each with a different sequence of "Stop type" entries, from which the network spaces are derived.

Scenario	Network	Space	Module	N_m	Module Flow
	DSN	Stations	1	396	0.82
	DSN	Stations	2	83	0.09
	DSN	Stations	3	151	0.09
	DSN	Stops	1	112	0.70
	DSN	Stops	2	19	0.09
	DSN	Stops	3	38	0.07
	DSN	Stops	4	22	0.05
	DSN	Stops	5	44	0.03
	DSN	Stops	6	22	0.02
	DSN	Stops	7	25	0.02
	DSN	Stops	8	26	0.01
	DSN	Stops	9	23	0.01
	DSN	Changes	1	200	0.79
R24	DSN	Changes	2	87	0.12
	DSN	Changes	3	44	0.09
	DTN	Stations	1	255	0.45
	DTN	Stations	2	82	0.20
	DTN	Stations	3	107	0.17
	DTN	Stations	4	118	0.12
	DTN	Stations	5	68	0.07
	DTN	Stops	1	188	0.70
	DTN	Stops	2	112	0.21
	DTN	Stops	3	31	0.09
	DTN	Changes	1	138	0.59
	DTN	Changes	2	87	0.19
	DTN	Changes	3	40	0.10
	DTN	Changes	4	44	0.10
	DTN	Changes	5	22	0.02
	DSN	Stations	1	361	0.82
	DSN	Stations	2	180	0.09
	DSN	Stations	3	92	0.09
	DSN	Stops	1	187	0.80
	DSN	Stops	2	49	0.10
	DSN	Stops	3	99	0.10
	DSN	Changes	1	186	0.81
	DSN	Changes	2	99	0.11
	DSN	Changes	3	50	0.08
R33	DTN	Stations	1	241	0.39
	DTN	Stations	2	109	0.23
	DTN	Stations	3	206	0.22
	DTN	Stations	4	77	0.16
	DTN	Stops	1	183	0.68
	DTN	Stops	2	109	0.20
	DTN	Stops	3	43	0.12
	DTN	Changes	1	165	0.64
	DTN	Changes	2	84	0.16
	DTN	Changes	3	45	0.11
	DTN	Changes	4	41	0.09

Table S2: **Norwegian Timetable; first Level of clustering results.** The modular structure was obtained by applying the Infomap clustering algorithm to weighted network representations, considering both the Directed Service Network (DSN) and the Directed Travel Time Network (DTN) across the Spaces of Stations, Stops, and Changes. In this first-level analysis, the primary communities (modules) are identified without delving into deeper hierarchical sub-structures, thereby capturing the most prominent partitions of the network based on service flows and travel time efficiencies. For each module, we report the number of associated nodes (N_m) and the corresponding normalized Module Flow, offering a quantitative characterization of the module's structural and functional relevance within the overall timetable network. This first-level clustering provides a foundational understanding of how connectivity is organized at the macroscopic scale across different spatial and operational dimensions.
