# Supplementary material for: Unveiling connectivity patterns of railway timetables through complex network theory and Infomap clustering
Source: PLoS One. 2025 Jul 18;20(7):e0328681. doi: 10.1371/journal.pone.0328681 (PMC12273951; doi:10.1371/journal.pone.0328681)
Supplement: Attachment — Submitted filename: Response to Reviewers.pdf [file pone.0328681.s002.pdf]

Response to Reviewers

PONE-D-25-21673
Unveiling connectivity patterns of railway timetables through complex network theory and Infomap clustering

Before entering into the technical discussion, we would like to thank all reviewers and the editor for the report. We really appreciate the effort that no doubt will contribute to significantly improve our manuscript. In the following document we reported in blue the Editor's and Reviewers' comments, and in *italic black* our responses/comments/integrations.

Journal Requirements:

1. When submitting your revision, we need you to address these additional requirements. Please ensure that your manuscript meets PLOS ONE's style requirements, including those for file naming. The PLOS ONE style templates can be found at:
https://journals.plos.org/plosone/s/file?id=wjVg/PLOSOne_formatting_sample_main_body.pdf
https://journals.plos.org/plosone/s/file?id=ba62/PLOSOne_formatting_sample_title_authors_affiliations.pdf

We corrected the format citation for Figures from "Figure x" to "Fig x" and fix the capitalization of paragraphs and section names.

2. We note you have included a table to which you do not refer in the text of your manuscript. Please ensure that you refer to Table 1 in your text; if accepted, production will need this reference to link the reader to the Table.

We realized that we put the Table 1 in a wrong position with respect to the citation; we moved Table 1 after the "Levels, flows and timetable characteristics" paragraph where it should belong, and put the correct citation appropriately.

Reviewer #1:

3. Quality of paper is very poor. Quality of picture, managing paper is very bad and resubmit. I suggest to retype paper with consulting professional writer. Please use
https://www.researchgate.net/publication/369618584_Standard_format_for_writing_paper_in_industrial_engineering_optimization_web_of_science_journal

Page size: A4 and 21*29.7
Header from top: 1.27 cm
Footer from bottom: 1.27 cm
Margin: normal 2.54 from left, right, top, bottom
Font: Times New Roman Size:12

Regarding the format, we wrote the manuscript over the Latex template provided by PLOS One; still, figures, captions and text have been re-checked again thanks to the Editor's and Reviewers' comments.

Reviewer #2:

4. Firstly, this manuscript investigates connectivity patterns in railway timetables using complex network theory and Infomap clustering, specifically applied to Norwegian railway scenarios for 2024

and projected conditions for 2033. The central objective, clearly articulated in the abstract (page 1), aims at understanding how service frequencies, travel times, and hierarchical structures affect overall network connectivity. The abstract is well-written, concise, and informative, although I suggest explicitly mentioning the key comparative insights between the two analyzed scenarios for greater impact.

We added a sentence in the abstract that better define the differences in connectivity in the scenarios.

5. The introduction (pages 1-2, lines 1-60) effectively sets the research context, outlining key concepts such as connectivity, service optimization, and robustness in railway timetabling. However, the section could significantly benefit from a more explicit statement regarding the novelty and contributions of this specific study, differentiating it clearly from previous research mentioned on lines 12-16 (papers [1]-[4]). Cite this "By examining these factors, researchers aim to develop schedules that minimize delays, improve synchronization between trains, and ensure seamless passenger transfers" as https://doi.org/10.1109/ICIRT.2016.7588740 . Cite this as "Complex networks are defined as systems composed of many elements (nodes and edges) interacting with each other." as https://doi.org/10.1016/j.ress.2021.108130 . While relevant studies are referenced, a succinct paragraph clearly articulating this study's unique methodological contribution—particularly the application of Infomap clustering—is strongly advised for clarity.

We have added the paper by Osman et al. (2016) as an appropriate citation in the introduction. Although the work by Dindar et al. (2022) explores "complex" modelling within railway operations, our original intention in stating that "Complex networks are defined as systems composed of many elements (nodes and edges) interacting with each other" was to refer to the broader field of Complex Network Theory, rather than to operational modelling per se. Nonetheless, we agree that the Dindar et al. (2022) paper is relevant and have cited it in the introduction where we mention: "[…] Advanced techniques, including mathematical modelling and simulation, are used to evaluate different timetable scenarios, ultimately guiding decisions that improve overall service quality and operational efficiency in railway systems," as it more directly pertains to modelling operational efficiency.

We have also added the requested paragraph that highlights the novelty of our contribution.

6. The methodological approach (pages 2-6, lines 61-260) is comprehensive and robust. The authors explain their three distinct "Spaces" (Stops, Stations, Changes) clearly. The synthetic model explanation on page 3 (lines 82-108) is well-structured and helpful in clarifying the methodological process. Figure 1 (page 3) is particularly effective in illustrating the conceptual differences among the three "Spaces". However, the caption of Figure 1 lacks details explaining colors or specific node labels within the figure; this should be improved for immediate clarity.

We updated the caption of Figure 1 as requested; we also slightly update Figure 1 adding some more legends for better readability and consistency with the updated caption.

7. In the section describing the transformation of timetables into weighted graphs (lines 109-127), the distinction between Directed Service Network (DSN) and Directed Travel Time Network (DTN) is clear. However, I recommend explicitly defining how exactly the travel time weights were computed—whether the inverse travel time metric used is linear or otherwise weighted should be made explicit. This point currently lacks sufficient detail.

We have clarified the methodology used to compute the travel time weights in the Directed Travel Time Network (DTN). Specifically, we have added the following sentence to the corresponding section (lines 138-141 of the updated manuscript):

"In the DTN, the weight assigned to each directed edge is computed as the inverse of the average travel time t between two nodes across all relevant services. This inverse is applied linearly (i.e.,

w=1/t), so that faster connections yield higher weights, emphasizing their greater contribution to network performance."

This clarification ensures that the reader understands the logic behind using inverse travel times and confirms that the transformation is linear and unweighted unless otherwise specified.

8. Regarding the Infomap clustering description (lines 128-156), the manuscript adequately explains why Infomap is appropriate. Still, I recommend briefly mentioning why Infomap was chosen over other popular clustering algorithms (e.g., Louvain or Leiden), ensuring transparency of methodological choice.

We have added the following sentence at the end of the Infomap clustering description (lines 177-183 of the update manuscript):

"We selected Infomap over other popular clustering algorithms such as Louvain or Leiden because it is particularly well-suited for flow-based, directed, and weighted networks—characteristics that align closely with our representation of timetable connectivity. Unlike modularity-based methods, Infomap leverages information theory to capture dynamic flow structures, making it especially effective in identifying communities in transportation networks where edge direction and flows (e.g., service frequency or travel time) are essential."

9. Table 1 (page 5) is clearly presented but could be better integrated with the surrounding explanatory text, directly interpreting the significance of the module flow and node-level flow values provided in practical terms.

We have added the following text to Table 1 caption, changing also the name of the table column "Flow" to "Node-Level Flow" for better interpretability:

"In practical terms, the Module Flow quantifies the relative importance of each cluster within the network based on the cumulative flow of services it handles. A higher module flow indicates that the corresponding cluster of stations plays a central role in service distribution. Similarly, Node-Level Flow values reflect how critical individual stations are within their modules: for example, Station B, with the highest Node-Level Flow, acts as a major interchange or hub, supporting both regional and express services. These values provide a quantitative insight into the hierarchy of service importance, helping to identify core nodes for operational planning and resilience analysis."

This addition aims to enhance the interpretability of Table 1 and underscore its practical relevance for understanding network structure.

10. The "Timetable Connectivity Index" (Tc), introduced on pages 6-7 (lines 213-260), is a valuable innovation. The illustrative example in Figure 3 (page 7) effectively demonstrates the practical implications of this metric. However, scenario "d" in Figure 3, involving the increased weight from 1 to 10, requires a clearer textual explanation (lines 250-256) to illustrate why the increase in connectivity is described as marginal despite the substantial numeric change in the number of services.

We have clarified (lines 283-291 of the updated manuscript) why the Timetable Connectivity Index (Tc) increases only marginally despite the significant increase in service frequency. The revised text emphasizes that Tc reflects structural changes in modular connectivity, and that additional services which do not alter the modular configuration yield limited gains in connectivity as measured by Tc.

11. The Results section (pages 7-10, lines 261-410) provides robust empirical insights. Figure 4 (page 8) clearly demonstrates the spatial differences between the three Spaces. Yet, it lacks specific descriptions or a more detailed legend clarifying the meaning of node size or arrow thickness explicitly, and this should be improved to enhance readability.

The purpose of Figure 4 was to highlight the spatial differences across the three network Spaces (Stops, Stations, and Changes), rather than to convey weighted or directional information. At this stage of the analysis, node and edge sizes are uniform across all Spaces, as they do not yet encode weights or flows. To address the reviewer's comment, we have improved both the figure and its caption. The revised version now includes annotations for major hubs (e.g., Oslo, Bergen, Trondheim, Stavanger) and clarifies the types of edges—such as shortcuts in the Space of Stops. Additionally, we have updated the caption to explicitly state that darker areas in the visualization indicate spatial clustering of nodes, often corresponding to urban hubs or high station density.

12. Figures 5 and 6 (pages 9-10) comparing the Directed Service Network (DSN) and Directed Travel Time Network (DTN) scenarios for R24 and R33 provide crucial insights. However, captions are insufficiently detailed, particularly lacking explicit interpretative statements of what changes between scenarios imply for railway connectivity and operational planning.

We have substantially revised the captions to include more detailed interpretative statements. The updated captions now explicitly describe how variations in the Timetable Connectivity Index (Tc) across the R24 and R33 scenarios reflect differences in service distribution, infrastructure, and travel time efficiency. We also highlight the operational planning implications—such as the impact of new services on regional accessibility or the role of hubs in network cohesion.

13. The textual discussion (lines 290-410) clearly identifies critical changes between 2024 and 2033 but occasionally lacks precise numerical evidence when stating increases or decreases in Tc values. I strongly recommend that these numerical changes are explicitly stated in parentheses next to qualitative claims (e.g., "increase from 0.28 to 0.30") to improve clarity and reinforce analytical rigor.

We have reviewed the Results section and reinforced all qualitative statements regarding changes in Timetable Connectivity Index (Tc) values by explicitly including the corresponding increase/decrease numerical changes, wherever they were not already stated.

14. Regarding "Scope and Limitations" (lines 411-436), the authors appropriately acknowledge limitations related to the static nature of the data and the exclusion of reliability metrics. Nevertheless, explicitly suggesting potential methodologies to incorporate reliability measures or robustness indicators in future studies would significantly strengthen the discussion.

To maintain coherence and avoid overextending the existing "Scope and Limitations" section (according to other reviewer's comment, see point 19) we have added a dedicated section titled "Future work and framework extensions", which introduces methodological directions for integrating reliability and robustness into our approach.
Specifically, we acknowledge that while our current study is based on static timetable data, the proposed framework can be extended to incorporate real-time or stochastic travel time data. This would enable the construction of a Robustness Travel Time Network (RTN), where edge weights reflect empirical or simulated delay scenarios, thus supporting robustness analysis from both the operator's perspective (e.g., buffer time margins) and the passenger's perspective (e.g., transfer penalties, waiting time, number of interchanges). The robustness index could be defined as the expected increase in passenger disutility under sampled delay distributions, following methodologies similar to those suggested by Takeuchi et al. (2007).
Incorporating formal reliability metrics, such as the Scheduled Speed Heterogeneity Ratio (SSHR) and Scheduled Arrival Headway Regularity (SAHR) proposed by Vromans et al. (2006), could improve the framework's sensitivity to operational irregularities.
As our clustering framework is designed to detect structural patterns across various network abstractions (Spaces), we believe that applying it to these extended RTN-type networks will allow the identification of vulnerability zones—i.e., areas where the structural timetable design leads to diminished reliability or robustness in practice. These directions, once tested, represent a promising way for expanding the practical utility of our method in both planning and monitoring contexts.
We summarized the above concepts into the "Future work and framework extensions" section (lines 482-503 of the update manuscript).

15. The Conclusion section (lines 437-462) succinctly summarizes the key findings but lacks explicit mention of concrete implications for practice or specific recommendations for railway operators. I advise adding a paragraph explicitly linking study results to practical timetable improvements, enhancing the applied relevance of the paper.

We have added a final paragraph to the Conclusion section that explicitly links our findings to practical applications in railway operations. The new paragraph highlights how the Timetable Connectivity Index (Tc) and modular analysis can support railway operators in optimizing timetable design, especially in future planning scenarios. The Timetable Connectivity Index framework might also find place within timetabling software and tools, adding values to developers and users aiming to quantitative compare different timetable structures.

16. Finally, I examined references (pages 12-14, lines 1-14). Overall, references are appropriately selected and appear relevant. However, consistency in citation formatting needs substantial improvement to comply with journal guidelines. Several references lack in literature review (I suggest the two: please look above, check all lines to see any place requiring for being cited) and some references lack DOI numbers (for example, references [1]) and uniform formatting (reference [10] missing page ranges), requiring thorough rechecking and revision.

We have thoroughly reviewed all references to ensure alignment with PLOS ONE's citation style, as specified by the plos2015.bst BibTeX style sheet available from PLOS One. We used it within the official Latex Template to write and format the full paper.

In particular:

- *Reference [10] in the original manuscript does include the correct page ranges (as formatted by Phys. Rev. E Journal), and no changes were required.*
- *The MapEquation citation refers to a software package, and we followed the citation format recommended by the developers on their official site which does not include a DOI.*
- *Regarding DOI numbers in conference proceedings (e.g., Reference [1] in the original manuscript), we note that PLOS style omits DOI information for proceedings-type publications, consistent with the formatting automatically generated using plos2015.bst.*

Finally, we carefully reviewed all lines in the manuscript to ensure that the two previously suggested references (Osman et al., 2016; Dindar et al., 2022) are cited in appropriate locations within the Introduction and supported by the updated discussion on modelling approaches and network theory. We also added some more consistency in citations across all introduction section, including more references related to complex network theory, timetable robustness and indicators of performance. Together with the already cited references, we added to the paper the following citations addressing also other reviewers' comments (see point 23):

- *Solinen E, Palmqvist CW. Development of new railway timetabling rules for increased robustness. Transport Policy. 2023;133:198–208. doi:10.1016/j.tranpol.2023.02.003.*
- *Vromans MJCM, Dekker R, Kroon LG. Reliability and heterogeneity of railway services. European Journal of Operational Research. 2006;172(2):647–665. doi:10.1016/j.ejor.2004.10.010.*
- *Takeuchi Y, Tomii N, Hirai C. Evaluation Method of Robustness for Train Schedules. Quarterly Report of RTRI. 2007;48(4):197–201. doi:10.2219/rtriqr.48.197.*
- *Von Ferber C, Holovatch T, Holovatch Y, Palchykov V. Public transport*

networks: empirical analysis and modeling. The European Physical Journal B. 2009;68(2):261–275. doi:10.1140/epjb/e2009-00090-x.

- *Newman MEJ. The Structure and Function of Complex Networks. SIAM Review. 2003;45(2):167–256. doi:10.1137/S003614450342480.*

- *Bagler G. Analysis of the airport network of India as a complex weighted network. Physica A: Statistical Mechanics and its Applications. 2008;387(12):2972–2980. doi:10.1016/j.physa.2008.01.077.*

- *Wang LN, Wang K, Shen JL. Weighted complex networks in urban public transportation: Modeling and testing. Physica A: Statistical Mechanics and its Applications. 2020;545:123498. doi:10.1016/j.physa.2019.123498.*

- *Nadinta DS, Surjandari I, Laoh E. A Clustering-based Approach for Reorganizing Bus Route on Bus Rapid Transit System. In: 2019 16th International Conference on Service Systems and Service Management (ICSSSM); 2019. p. 1–6.*

- *Zhong C, Arisona SM, Huang X, Batty M, and GS. Detecting the dynamics of urban structure through spatial network analysis. International Journal of Geographical Information Science. 2014;28(11):2178–2199. doi:10.1080/13658816.2014.914521.*

17. In conclusion, I believe that this manuscript presents a valuable methodological innovation in railway timetable analysis with clear practical potential. The strengths include a solid conceptual framework, effective use of Infomap clustering, and insightful empirical analysis comparing different future scenarios. Critical improvements required for this manuscript involve clearer explicit articulation of the novelty, improved methodological detail (especially weights and Infomap choice), more precise and informative figure/table annotations, explicit numerical references to enhance analytical clarity, and stringent adherence to citation standards. These substantial improvements will significantly enhance the manuscript's clarity, methodological rigor, and applicability, making it a robust contribution to transportation systems research.

We sincerely thank the reviewer for their thoughtful evaluation and constructive summary. In response to the valuable suggestions provided, we have made substantial revisions throughout the manuscript to enhance clarity, methodological transparency, and applied relevance.
We believe these improvements have significantly strengthened the manuscript and hope it now meets the expectations for publication in PLOS ONE.

Reviewer #3:

The article is well drafted, and to the point, however I have certain suggestions for the authors:

18. The abstract and the conclusion can be improved by adding some numbers from the results.

We have updated the Conclusion section to highlight key numerical results, specifically noting the increase in Tc within the Space of Stops from 0.26 in R24 to 0.49 in R33. As for the Abstract, it has been revised in response to another reviewer's comment (see point 4). While we acknowledge the suggestion to include numerical values in the Abstract, we believe that maintaining a concise, qualitative summary allows for better contextual interpretation after reading the full discussion. For this reason, we opted not to include specific numbers in the Abstract.

19. The Scope and Limitations section can be narrowed down.

We tried to synthetize the Scope and Limitations section, moving some old and new information in the new “Future work and framework extensions” section, compatibly to other reviewers’ comments and requests about this section (see point 14 and point 22).

20. The authors can add some data in form of a table to make it easier for the readers to understand what they are working on.

We believe that Table S1 in Supporting Information is informative about the timetable structure we used as input data to the full framework. Table S2 in Supporting Information also represent the full output provided by the tool in terms of Module Flows.

Reviewer #4:

Thank you for the submission. The study introduces a useful framework combining complex network theory with Infomap clustering to analyze railway timetables. The Timetable Connectivity Index (Tc) is an interesting contribution, and the real-world application to Norwegian scenarios adds practical relevance.

21. The paper would benefit from more clarity on practical applicability. How would this metric inform real-world decisions by operators or planners? Could it integrate into existing scheduling or diagnostic tools?

Practical applicability information about this framework have been included in the Conclusions section (see also point 15). The Timetable Connectivity Index (Tc) could be integrated into existing scheduling and diagnostic platforms, such as Treno by Trenolab, which already support timetable scenario evaluation and simulation. By embedding Tc into these workflows, operators could supplement traditional KPIs (e.g., punctuality, dwell time) with structural insights on how changes affect modular connectivity, supporting scenario comparison within the platform.

22. Please also expand on the transferability of the framework. Would this approach remain effective in larger, denser, or multimodal networks? A discussion on its limitations or adaptability beyond the Norwegian context would add value.

Regarding the scale, the Timetable Connectivity Index is already normalized by the total number of nodes in the networks (see point 26), so that we can use it for comparisons across networks of different sizes. However, comparability is most meaningful when the underlying assumptions—such as the selected network space, weighting method (DSN or DTN), and modular level—are consistent across scenarios or systems.
Regarding multimodal applicability, while the Infomap algorithm can support multilayer and multiplex networks, we believe that applying the current framework to multimodal systems introduces additional complexity. In particular, integrating different transport modes (e.g., metro, bus, tram) would require harmonizing input metrics such as service frequency and travel time, which often operate at different temporal and spatial scales. Moreover, proper modeling of intermodal transfers, including penalties, synchronization rules, and walking times, would be essential to preserve interpretability and accuracy. Still, we believe the framework provides a solid foundation for extension to more complex systems. Future research could explore how to adapt the methodology for multimodal networks by incorporating layer-specific weights and intermodal transfer modeling.
We summarized the above concepts into the “Future work and framework extensions” section (lines 482-503 of the update manuscript).

23. The literature review should be broadened. More recent studies on clustering in transit systems, robustness in timetables, and alternative performance metrics would better situate this work within current research.

We have broadened the literature review to include additional recent studies related to clustering in transit systems, timetable robustness, and alternative performance metrics. These references were carefully selected to align with the scope and methodological focus of our paper, and they help better situate our contribution within the current body of research. The expanded discussion now highlights complementary approaches in network-based transit analysis and clarifies the novelty of our flow-based connectivity framework.

We added the following new suggested references (see point 5) and new ones to address points raised in point 16:

- *Bin Osman MH, Kaewunruen S, An M, Dindar S. Disruption: A new component in the track inspection schedule. In: 2016 IEEE International Conference on Intelligent Rail Transportation (ICIRT); 2016. p. 249–253.*
- *Dindar S, Kaewunruen S, An M. A hierarchical Bayesian-based model for hazard analysis of climate effect on failures of railway turnout components. Reliability Engineering and System Safety. 2022;218:108130. doi:10.1016/j.ress.2021.108130.*
- *Newman MEJ. The Structure and Function of Complex Networks. SIAM Review. 2003;45(2):167–256. doi:10.1137/S003614450342480.*
- *Bagler G. Analysis of the airport network of India as a complex weighted network. Physica A: Statistical Mechanics and its Applications. 2008;387(12):2972–2980. doi:10.1016/j.physa.2008.01.077.*
- *Wang LN, Wang K, Shen JL. Weighted complex networks in urban public transportation: Modeling and testing. Physica A: Statistical Mechanics and its Applications. 2020;545:123498. doi:10.1016/j.physa.2019.123498.*

We added the following new reference about timetable robustness and reliability analyses:

- *Solinen E, Palmqvist CW. Development of new railway timetabling rules for increased robustness. Transport Policy. 2023;133:198–208. doi:10.1016/j.tranpol.2023.02.003.*
- *Takeuchi Y, Tomii N, Hirai C. Evaluation Method of Robustness for Train Schedules. Quarterly Report of RTRI. 2007;48(4):197–201. doi:10.2219/rtriqr.48.197.*

We added the following new reference about alternative performance metrics:

- *Von Ferber C, Holovatch T, Holovatch Y, Palchykov V. Public transport networks: empirical analysis and modeling. The European Physical Journal B. 2009;68(2):261–275. doi:10.1140/epjb/e2009-00090-x.*
- *Vromans MJCM, Dekker R, Kroon LG. Reliability and heterogeneity of railway services. European Journal of Operational Research. 2006;172(2):647–665. doi:10.1016/j.ejor.2004.10.010.*

We added the following new references about clustering in transit systems:

- *Nadinta DS, Surjandari I, Laoh E. A Clustering-based Approach for Reorganizing Bus Route on Bus Rapid Transit System. In: 2019 16th International Conference on Service Systems and Service Management (ICSSSM); 2019. p. 1–6.*

- *Zhong C, Arisona SM, Huang X, Batty M, and GS. Detecting the dynamics of urban structure through spatial network analysis. International Journal of Geographical Information Science. 2014;28(11):2178–2199. doi:10.1080/13658816.2014.914521.*

A few brief questions for the authors:

24. Why was Infomap chosen over other clustering methods?

We have added the following sentence at the end of the Infomap clustering description (lines 177-183 of the update manuscript):

"We selected Infomap over other popular clustering algorithms such as Louvain or Leiden because it is particularly well-suited for flow-based, directed, and weighted networks—characteristics that align closely with our representation of timetable connectivity. Unlike modularity-based methods, Infomap leverages information theory to capture dynamic flow structures, making it especially effective in identifying communities in transportation networks where edge direction and flows are essential."

25. How sensitive is Tc to different time windows (e.g., peak vs. off-peak)?

To measure the sensitivity of the Timetable Connectivity Index over different time windows (in the manuscript we analysed a typical full day of operations only), we had to perform new analyses filtering out peak and off-peak time frames.
The first thing to do is to define peak and off-peak periods in our Norwegian timetable. Since we are dealing with services at a national scale, we didn't expect particular differences between peak and off-peak services in terms of number of trains running (with the exception of nighttime services). Still, thanks to the histograms in S3 Fig. in Supporting Information, we were able to define two peaks (one in the morning between 7 and 8, one in the afternoon between 16 and 17). Those peaks are consistent in both scenarios (R24 and R33) despite the higher number of trains running in the forecasted timetable. We verified also the distribution among arrival times, and they are consistent with the previously defined peak time frames. We then filter out our initial dataset getting information related to train departing in peak only hours and off-peak only hours, applying our general framework on the filtered datasets.
The comparison between Tc values across different time windows does reveal important insights into the temporal dynamics of railway timetable connectivity. S3 Fig. shows the average value of Tc over Full Day, Peak and Off-Peak periods for all the Space of analyses, in the DSN c) and DTN d) network, along with the standard deviation variability.
For both networks, Tc during peak periods is generally higher than in the full-day and off-peak time frames. This is due to the more concentrated services during peak hours, which typically form well-defined clusters—both in terms of service frequency and travel time. In the DSN network, Tc is higher in the forecasted scenario (R33) when considering the full-day period. However, peak and off-peak values are lower compared to the current R24 timetable. This suggests a redistribution of services in R33 that favors peak operations and potentially prioritizes express routes, leading to reduced modular cohesion during off-peak periods. The decline in off-peak Tc may reflect a fragmentation of stop-to-stop connectivity for local services in the future timetable. Less variability is observed between the two scenarios in the DTN network, where—despite the inclusion of faster and more frequent long-distance services in R33—the average travel time-based accessibility at the stop level declines, likely due to express services bypassing intermediate stops.
These results highlight that Tc is not only structurally but also temporally sensitive—capable of detecting how connectivity strength shifts across service windows. This temporal granularity:

- *Enables planners to pinpoint periods of weak connectivity and assess where service adjustments may be needed.*
- *Underscores the value of scenario-specific analysis, as the future-oriented R33 timetable exhibits different temporal performance characteristics compared to R24.*

We summarized the above results in the updated manuscript in lines 449-456 of the updated manuscript, adding explicative figures in S3 Fig. in the updated Supporting Information document.

26. Is Tc comparable across networks of different sizes, or does it require normalization?

We added a paragraph (lines 295-299 of the update manuscript) clarifying that the Tc index is normalized by the total number of nodes in the network and ranges from 0 to 1, so that we can use it for comparisons across networks of different sizes. However, comparability is most meaningful when the underlying assumptions—such as the selected network space, weighting method (DSN or DTN), and modular level—are consistent across scenarios or systems.

27. I recommend revision to address these points. With clearer context, broader references, and practical discussion, the manuscript will make a strong contribution.

Thank you again for the comments and the insightful points raised during the revision process. We hope that we have addressed all the issues raised and that the manuscript is now more complete and clearer.

---

28. While revising your submission, please upload your figure files to the Preflight Analysis and Conversion Engine (PACE) digital diagnostic tool, https://pacev2.apexcovantage.com/. PACE helps ensure that figures meet PLOS requirements. To use PACE, you must first register as a user. Registration is free. Then, login and navigate to the UPLOAD tab, where you will find detailed instructions on how to use the tool. If you encounter any issues or have any questions when using PACE, please email PLOS at figures@plos.org. Please note that Supporting Information files do not need this step.

We successfully checked figures with PACE as requested and downloaded the PACE corrected .tif files (see confirmation screenshot), inspecting figures for image clarity and content too.

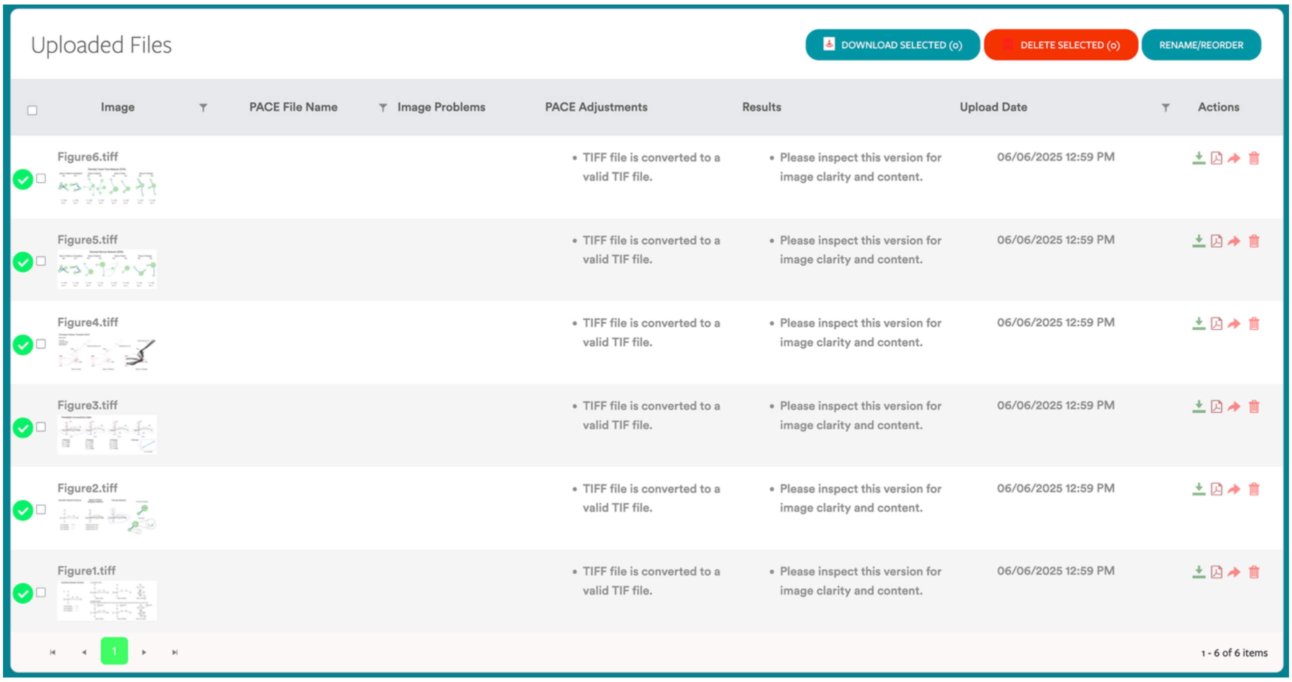

We have carried out too a revision of the text in search for typos and grammatical errors. As a final remark, we would like to thank again reviewers and editor for the detailed report, and we hope that the manuscript is now suitable for publication.
